# Bellymount-pulsed tracking: a novel approach for real-time in vivo imaging of *Drosophila* abdominal tissues

**DOI:** 10.1093/g3journal/jkae271

**Published:** 2024-11-18

**Authors:** Shruthi Balachandra, Amanda A Amodeo

**Affiliations:** Department of Biological Sciences, Dartmouth College, Hanover, NH 03755, USA; Department of Biological Sciences, Dartmouth College, Hanover, NH 03755, USA

**Keywords:** oogenesis, in vivo live imaging, developmental dynamics, protein accumulation

## Abstract

Quantitative live imaging is a valuable tool that offers insights into cellular dynamics. However, many fundamental biological processes are incompatible with current live-imaging modalities. *Drosophila* oogenesis is a well-studied system that has provided molecular insights into a range of cellular and developmental processes. The length of the oogenesis, coupled with the requirement for inputs from multiple tissues, has made long-term culture challenging. Here, we have developed Bellymount-pulsed tracking (Bellymount-PT), which allows continuous, noninvasive live imaging of *Drosophila* oogenesis inside the female abdomen for up to 16 h. Bellymount-PT improves upon the existing Bellymount technique by adding pulsed anesthesia with periods of feeding that support the long-term survival of flies during imaging. Using Bellymount-PT, we measure key events of oogenesis, including egg chamber growth, yolk uptake, and transfer of specific proteins to the oocyte during nurse cell dumping with high spatiotemporal precision within the abdomen of a live female.

## Introduction

Advances in live imaging have been essential for unraveling the dynamics of key molecular events during a variety of biological processes. However, not all processes are equally amenable to live imaging. Capturing very small or rapid molecular events requires sophisticated microscopy (Fuhrmann *et al*. 2022). Conversely, processes occurring over longer timescales, such as growth and development, require preserving tissue health during long-term imaging (Morris and Spradling 2011; Peters and Berg 2016; Tsao *et al*. 2016; Martin *et al*. 2018). *Drosophila* oogenesis is one process that has proven to be resistant to long-term live imaging despite a wealth of genetic tools and molecular characterization (King 1970; Spradling 1993; Morris and Lehmann 1999; Shimada *et al*. 2011; Avilés-Pagán *et al*. 2020; Imran Alsous *et al*. 2021; Lebo and McCall 2021; Jackson *et al*. 2024). The major challenge in capturing the journey from a germline stem cell (GSC) to a fertilizable egg is that the process lasts ∼7 days and happens inside the female abdomen (He *et al*. 2011). The GSCs reside in the germarium at the anterior tip of the ovary. Differentiating cells leave the stem cell niche and undergo 4 rounds of incomplete mitosis to generate a 16-cell cyst. One cell is specified as the oocyte and the rest become nurse cells, which will endocycle to produce the bulk of the materials required for oocyte growth. The 16 germline cells are encapsulated by a layer of somatic follicle cells to form an egg chamber. The egg chamber exists the germarium and grows several orders of magnitude in volume while undergoing a stereotyped procession of developmental milestones over a period of ∼3 days (Dapples and King 1970; King 1970; He *et al*. 2011; Diegmiller *et al*. 2021). The developing egg chambers migrate posteriorly down the length of the ovary following one another in an assembly line called an ovariole. Each ovariole contains 5–7 egg chambers at any given time and a pair of ovaries will have 32–45 ovarioles (David and Merle 1968; King 1970).

Historically, most studies of *Drosophila* oogenesis have been performed using fixed samples, which necessarily limit the ability to resolve dynamic processes (King 1970; Spradling 1993; Diegmiller *et al*. 2021; Imran Alsous *et al*. 2021; Weichselberger *et al*. 2022). However, as early as the 1970s, efforts have been made to develop ex vivo culture protocols for late-stage egg chambers (Petri *et al*. 1979; Gutzeit and Koppa 1982). Optimization of late-stage cultures allows survival of stage 10–14 egg chambers for up to 10 h (Peters and Berg 2016; Parsons *et al*. 2023; Zhang and Liu 2024). Stage 9 and earlier egg chambers have proven to be more difficult to culture and require the addition of insulin to the media to achieve survival for 4–5 h (Prasad and Montell 2007). Further refinement of the culture media allowed for the survival of the germarium to a maximum of 13 h with follicle cell division rates declining after 6–8 h (Morris and Spradling 2011). Nevertheless, the specific growth rates in these culture conditions have not been reported and to date, many live-imaging studies are limited to only a few hour before egg chambers stall their growth and begin dying (Inaki *et al*. 2022; Zajac *et al*. 2023). Collectively, these techniques allow a short window to observe processes, including the germline-soma coordination in the germarium (Morris and Spradling 2011; Wilcockson and Ashe 2021), shuttling of biomolecules across the egg chamber (Cooley *et al*. 1992; Shimada *et al*. 2011; Lu and Gelfand 2022), follicle cell morphology and migration (Prasad and Montell 2007; Haigo and Bilder 2011; Cetera *et al*. 2014; Chen *et al*. 2017; Miao *et al*. 2022; Williams *et al*. 2022; Lei *et al*. 2023; Jackson *et al*. 2024), and nurse cell changes during later stages of oogenesis (Yalonetskaya *et al*. 2020; Imran Alsous *et al*. 2021; Jackson *et al*. 2024). Recently, Marchetti *et al*. developed a revolutionary multiorgan coculture system that successfully maintains egg chamber integrity for up to 3–4 days. However, growth rates remain substantially lower than in vivo and egg chambers fail to progress through developmental landmarks at the appropriate pace (Marchetti *et al*. 2022).

The recently developed Bellymount tool by Koyama *et al*. (2020) allows visualization of the internal structures, including the midgut, crop, intestinal bacteria, and ovaries without the need to open the fly's abdomen by affixing the fly between 2 glass coverslips using transparent, nontoxic Elmer's glue. In the original protocol flies were imaged over several days and were released from the mount in between image acquisitions. Though this technique captures unperturbed cellular dynamics in vivo with unprecedented resolution, the need to repeatedly capture and remount flies prevents tracking in tissues that lack morphological guideposts for image alignment. This is particularly problematic for oogenesis since each female has dozens of egg chambers, each growing rapidly and moving posteriorly during development. To overcome this challenge, we developed Bellymount-pulsed tracking (Bellymount-PT) by combining the original Bellymount restraint with pulsed anesthesia, and a liquid diet to allow long-term imaging and tracking of abdominal structures for periods up to 12–16 h. The prolonged imaging duration enabled us to track egg chamber growth and visualize transitions between developmental stages. Further, we were able to quantify aspects of yolk uptake and histone accumulation during nurse cell dumping, which have never been previously observed in an in vivo system. This study provides a novel technique for generating insight into the key events of oogenesis in an in vivo system with minimal experimental perturbation.

## Materials and methods

### Fly stocks

*Drosophila* stocks were maintained at room temperature (22 ± 2°C) on standard molasses corn fly media. The following fly stocks were used in this study: w; Mat-ɑ-tub67-gal4, Moesin-GFP/CyO; Mat-ɑ-tub15-gal4, H2Av-mRFP/TM3 (recombined line received as a gift from He Lab, Dartmouth College, NH, USA), w; Mat-ɑ-tub67-gal4/CyO; Mat-ɑ-tub15-gal4, H2Av-mRFP/TM3 (Wieschaus Lab, Princeton University, NJ, USA) (Hunter and Wieschaus 2000), yw; Sp/CyO; EndoH2Av-mCherry/TM3 (Shindo *et al*. 2024), and Yp1-sfGFP/CyO (VDRC, 318746) (Hara and Yamamoto 2022).

### Fly mount preparation

**Fig. 1.**
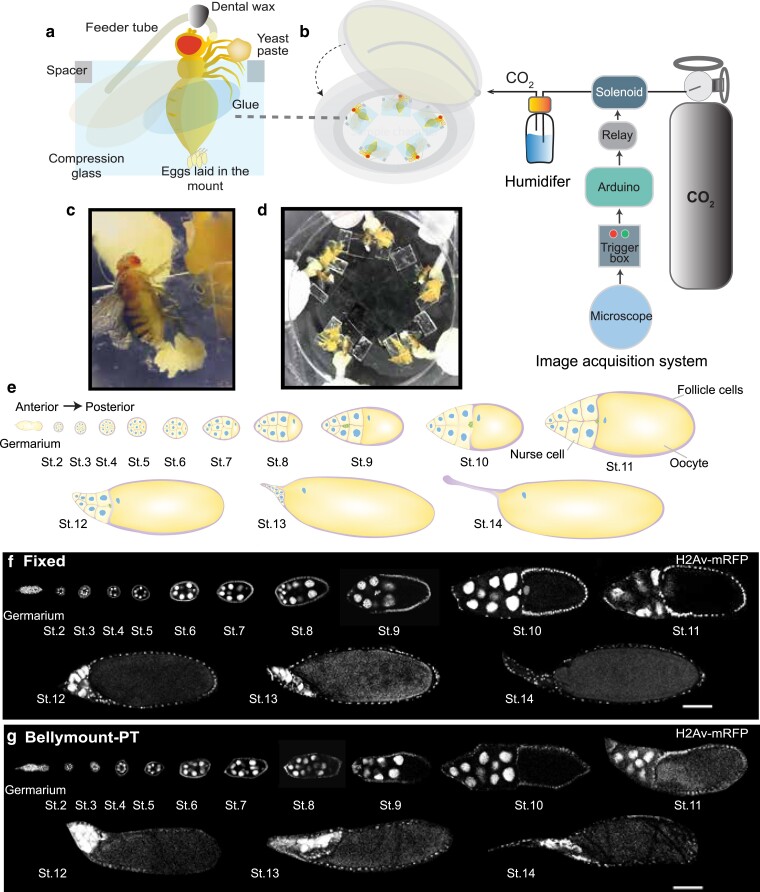
Bellymount-pulsed tracking (PT) allows long-term live imaging of oogenesis in live females. a) Schematic of a fly mounted for Bellymount-PT. Well-fed mated females were glued on the glass surface of a MatTek dish using Elmer's glue and compressed between the dish and the glass coverslip with 0.48 mm spacers to secure the fly during imaging. A small amount of fresh yeast paste and a feeder tube containing custom liquid apple juice, yeast extract food ensure proper nutrition. b) Image acquisition setup for confocal microscope and pulsed CO_2_ anesthetic. An Arduino programmed to regulate the CO_2_ flow via a solenoid value is connected to a signal from the microscope. CO_2_ is pulsed into the sample dish for 4 min during acquisition through a humidifying chamber. Flies are allowed to awaken and feed between time points. c) A female that remained alive and fecund after 16 h in the mount without imaging. Note the large number of eggs that have accumulated to the posterior of the fly. d) Whole mount with flies and feeder tubes. Five females can be imaged in the same dish simultaneously. e) Schematic showing oogenesis stages. The blue circles represent the nurse cells and the oocyte nuclei, the pink border represents the follicle cell layer, and the cells in green represent border cells. f) Staged fixed egg chambers expressing H2Av-mRFP from multiple females imaged at 20× (0.8 NA) on Zeiss LSM980 (scale bar = 100 µm). g) Collection of staged live egg chambers expressing H2Av-mRFP from multiple females acquired using Bellymount-PT imaging on the same microscope as Fig. 1f (scale bar = 100 µm). These egg chambers were imaged through the intact cuticle of the female abdomen. Adjacent egg chambers were cropped out and images were processed for presentation using background subtraction, Gaussian filter and median filter in Fiji.

The Bellymount imaging system was adapted from Koyama *et al*. (2020). A 4–5-day-old females raised on standard molasses corn fly food supplemented with fresh yeast paste were transferred to apple juice agar plates with fresh yeast paste 12–24 h before imaging. Healthy and well-fed females were anesthetized on a fly CO_2_ pad for mounting. A maximum of 5 females per experiment were mounted on the glass surface of a 50 mm MatTek dish (MatTeck, P50G-1.5-30-F) using clear Elmer's Liquid School Glue (Elmer's, E309). Adherence of the flies to the dish was accomplished as described in Koyama *et al*. (2020). A small drop of Elmer's glue was placed on the MatTeck dish near the edge of the glass surface. An anesthetized female was carefully placed on the glue and pressed gently with blunt forceps. The fly was secured by placing a 0.5 cm^2^ compression glass starting from her thorax (cut from a 24 × 60 mm cover glass; VWR International, 48393-106). A pair of 2 mm^2^ double-sided adhesive spacers of 0.48 mm thickness (MilliporeSigma, GBL620004-1EA) were placed on either side of the fly between the compression glass and the dish surface (Fig. 1a).

Flies were provided with a novel liquid food to keep them hydrated and well-fed during long-term imaging. A feeder tube was constructed by bending a 2 cm long glass disposable micropipette (VWR International, 53432-921) into a “J” shape using a Bunsen burner. This shape allowed a larger volume of liquid food to be provided to each fly within the space confines of the imaging dish. A wick made from cotton (Equate beauty, 681131169684) was inserted into the short end of the J to allow for the slow release of liquid media. Feeder tubes were filled with freshly made 0.5 mg/ml yeast extract (Thermo Fisher Scientific, BD 212750) in undiluted apple juice (Ocean Spray, 10031200007206). A few grains of active yeast (MP, 02101400-CF) were added to the liquid food, and the tip of the wick was dipped in banana baby food (Gerber, 12348015) immediately before mounting. The feeder tubes were secured with dental wax (Fresh Knight, 40201616938) adjacent to each fly's proboscis so they could feed during imaging intervals. Care was taken to position the feeder tube at an appropriate distance to avoid drowning the fly. A small dollop of yeast paste was provided near the flies’ legs to stimulate feeding.

### Pulsed anesthesia

For effective anesthetization and to maintain optimum humidity essential for long-term imaging, we covered the sample dish with a 50 mm petri dish lid (Fisher Scientific, 351007). We used this lid instead of the lid of the MatTek dish to increase the depth of the sample dish to allow the insertion of moistened absorbent pads (Hazmat Sorbent Pads, S-14748) cut to a diameter of 40 mm with a slit of 15 mm along the diameter and CO_2_ tubing. Both were secured with a metal mesh (Genesee Scientific, Flystuff 57–100 Mesh, Stainless Steel 97 µm) cut to 50 mm diameter. A hole was drilled on the side wall of the lid to allow entry of a 1/16″ diameter flexible plastic tube (MilliporeSigma, Z765228) for the administration of CO_2_ anesthesia. Through this hole, CO_2_ tubing was inserted and glued using KWIK-SIL adhesive silicone glue (World Precision Instruments, 60002) to the wall of the dish. 100% CO_2_ at 5 PSI (Airgas, AS215A320) was bubbled through distilled water in a 500-mL PeCon humidification bottle (PeCon) to increase humidity when CO_2_ was administered.

**Fig. 2.**
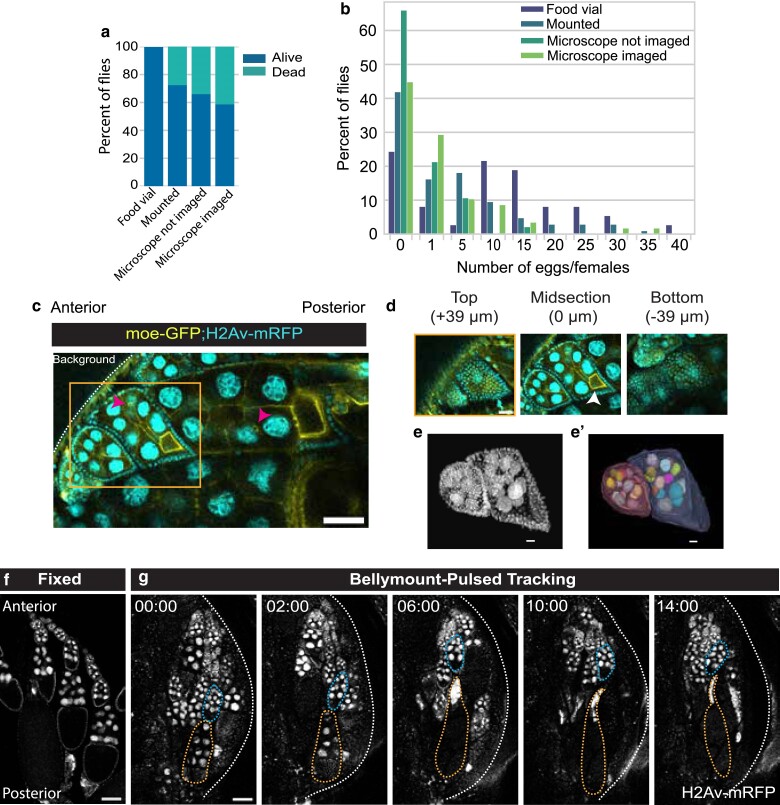
Pulsed anesthesia allows tracking for over 14 h. a) Survival percentage for females after 16 h of imaging compared with various controls. To understand the effect of mounting, anesthesia, and laser exposure we compared imaged flies (*n* = 58) to controls that were either never placed in the Bellymount-PT chamber (food vial, *n* = 37); mounted, but never exposed to the imaging protocol (mounted, *n* = 105), and those that were placed on the microscope during imaging, anesthetized but not exposed to laser (microscope, not imaged *n* = 47). Most of the observed mortality can be attributed to the restraints, though anesthesia and exposure to laser further decrease survival. b) Histogram of eggs laid in 16 h period for the same flies as in Fig. 2a. Females laid 0–40 eggs during the imaging period. Flies that remained in their food vials laid an average of 10 eggs per female, while flies imaged laid 2 eggs on average. c) Single time point representative image of multiple egg chambers within a single ovariole expressing H2Av-mRFP and Moesin-GFP using Bellymount-PT acquired on Olympus multiphoton microscope (25× water objective) (scale bar = 50 µm). d) All 3-cell types (oocyte, nurse, and follicle cells) can be visualized through the cuticle. There is sufficient *z-*resolution to ∼ 110 µm to capture the full volume of egg chambers stage 8 and younger. Pink arrowheads indicate ring canals and white indicates oocyte nucleus. Insets represent the top (+39 µm), midsection (0 µm), and bottom (−39 µm) *z-*slices of a stage 6 egg chamber (scale bar = 25 µm). (e,e’) 3D reconstruction of the egg chambers in Fig. 2d with nuclear H2Av-mRFP signal and the 3D constructed surface, respectively (scale bar = 10 µm). f) Fixed egg chambers with the same genotype and imaging as Fig. 2g for comparison. g) Live, developing egg chambers expressing H2Av-mRFP imaged over time using Bellymount-P (scale bar = 100 µm). Multiple egg chambers can be tracked in a single experiment. The egg chamber marked in blue starts in early stage 8 and increases in size over the imaging duration. The egg chamber marked in yellow starts in stage 10 and progresses through stage 14 with complete dorsal appendages formation visible by 14 h (scale bar = 100 µm). Dotted line separates the imaged fly abdomen and the background. Images in Fig. 1f–g were imaged on Zeiss LSM980 microscope with a 10× air objective. Images in Fig. 2c, d, f and g were processed for presentation using background subtraction, Gaussian filter and median filters in Fiji.

**Fig. 3.**
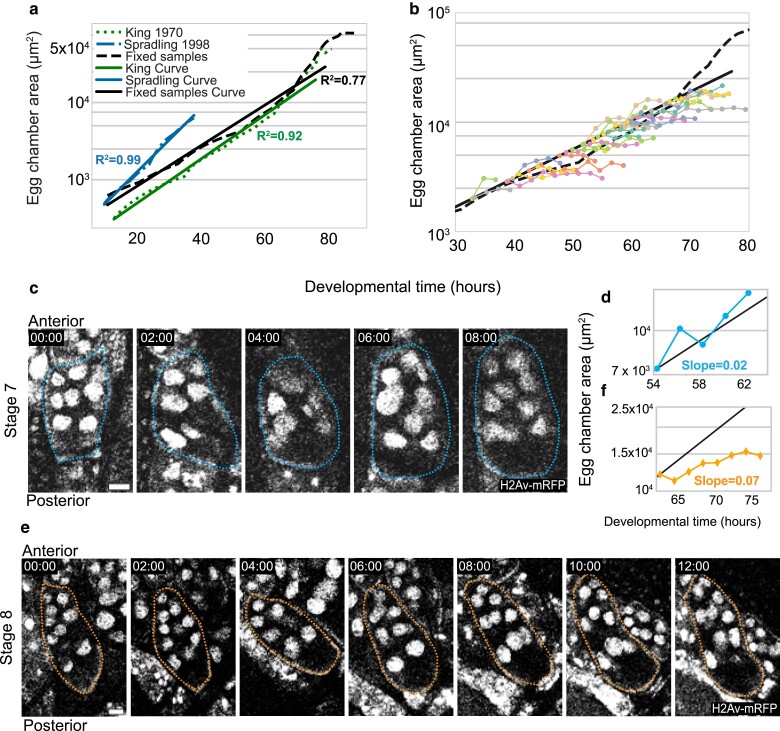
Bellymount-PT allows live monitoring of egg chamber growth. The midsection area of fixed egg chambers was used as a measure of egg chamber size. Fixed egg chambers were staged based on milestones of oogenesis as per (King 1970, see methods). The measured sizes combined with the frequency of each stage, total number of egg chambers per ovariole, and the number of eggs laid by individual females in 24 h were used to construct a standard growth curve (black line in panel a) for the fly line we used for live growth analysis (w; Mat-ɑ-tub67-gal4/CyO; Mat-ɑ-tub15-gal4, H2Av-mRFP/TM3). a) Comparison of standard growth curves from this study and previous literature derived from fixed samples. Egg chamber areas from King et al 1970 (green) and Spradling 1993 (blue) were used to generate an exponential standard growth curve along with the corresponding time estimates. *Y*-axis is in log scale. b) The midsection area of 38 egg chambers tracked using Bellymount-PT with 2 h imaging intervals. The resulting traces were aligned to the standard growth curve shown in Fig. 3b (black) by their initial area and with subsequent timepoints based on actual image acquisition times. Individual egg chambers were tracked for 4–18 h. *Y*-axis is in log scale. c) A previtellogenic egg chamber starting at stage 7 (scale bar = 25 µm). d) The change in midsection area over time for the egg chamber in Fig. 3c compared with the standard growth curve in black. Note the agreement between predicted and observed growth rates. *Y*-axis is in log scale. e) A vitellogenic stage 8 egg chamber tracked for ∼12 h (scale bar = 25 µm). f) The growth rate of the egg chamber in Fig. 3e compared with the standard curve. Note that growth is slower than predicted *y*-axis is in log scale. (c and e) were processed for presentation using background subtraction, Gaussian filter, and median filters in Fiji.

**Fig. 4.**
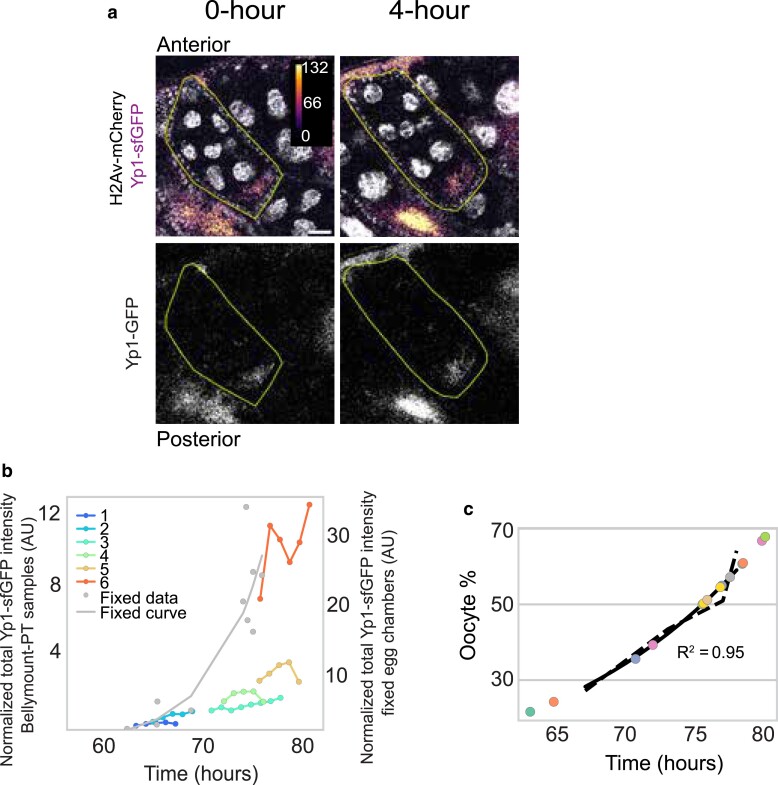
Bellymount-PT allows for the observation of dynamic events during oogenesis. a) Yolk protein accumulation during vitellogenic stages was tracked in flies expressing Yp1:sfGFP (scale bar = 25 µm). b) Quantification of the total Yp1:sfGFP intensity in the oocyte over time for 6 different egg chambers. We observe a steady increase in Yp1:sfGFP in all oocytes. Gray dots represent the Yp1:sfGFP from the fixed egg chamber used to generate the curve shown in gray plotted on an independent *y*-axis on the right (*n* = 12). (Egg chamber ages of both fixed samples and Bellymount-PT samples were estimated as in (Fig. 4c). c) A growth curve was generated based on the oocyte percent to estimate the age of the egg chambers (see methods and Supplementary Fig. 3b for correlation between egg chamber area and oocyte percent).

**Fig. 5.**
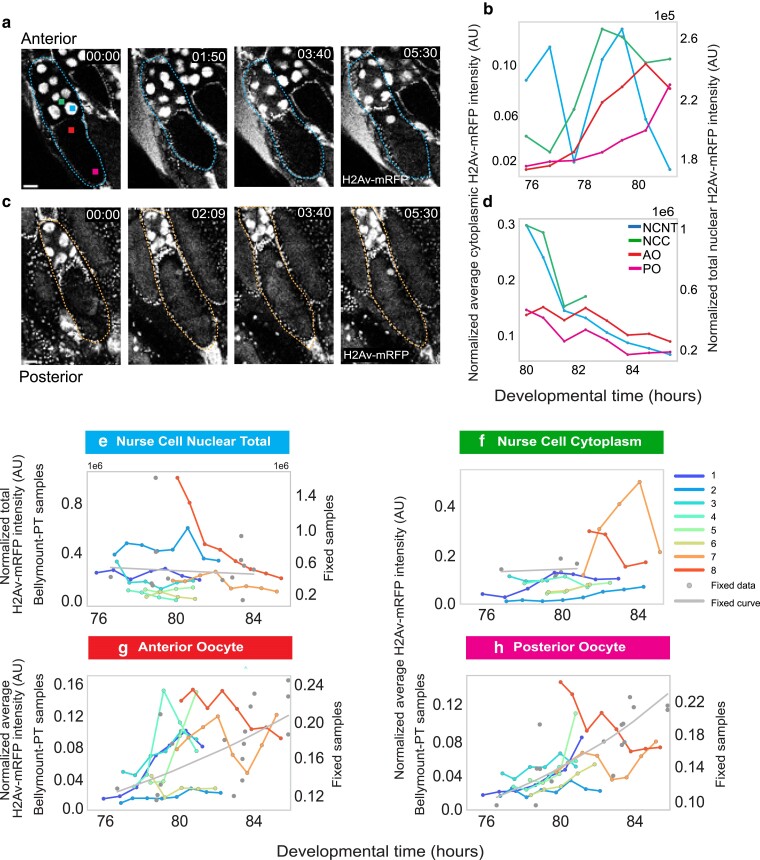
Maternal histone loading into the oocyte during nurse cell dumping in vivo. a) Stage 10 egg chamber expressing H2Av-mRFP during early nurse cell dumping. We observed an increase in H2Av in the nurse cell cytoplasm followed by an increase in the oocyte cytoplasm. Squares indicate the region of the egg chamber quantified in Fig. 5b and d–h (scale bar = 50 µm). b) Quantification of average pixel intensities in the indicated regions of the egg chamber shown in Fig. 5a normalized to the intensity of the follicle cell nuclei NCC-Nurse cell cytoplasm; AO, anterior oocyte cytoplasm; PO posterior oocyte cytoplasm along with the total intensity of the nurse cell nuclei (NCNT). NCNT is plotted on a separate *y*-scale on the right. Note the steady increase of H2Av in all 3 cytoplasmic regions as H2Av-mRFP exits the nurse cell nuclei (NCNT). (c) A more advanced, stage 11, egg chamber during later stages of dumping (scale bar = 50 µm). d) Quantification of the H2Av intensity in different regions of the egg chamber over time shown in Fig. 5c. Note that H2Av declines in all regions at these later stages. (e–h) Normalized H2Av intensities in various regions of 8 independent egg chambers of different ages. Each color corresponds to the same egg chamber in all panels. Egg chamber ages were estimated from the standard growth curve in Fig. 4c based on the percent of egg chamber area occupied by the oocyte at t0. Gray dots represent the intensities from fixed samples (*n* = 27), and the gray line is the curve generated from these fixed samples. Note the high degree of variability in the fixed data. Together these data are consistent with H2Av being lost from nurse cell nuclei and flowing gradually posteriorly into the oocyte. e) The total nurse nuclear intensities represent the total amount of H2Av in the nurse cell nuclei over time. Nurse cell nuclear concentrations can be found in Supplementary Fig. 4b. These intensities show a consistent decline over the course of nurse cell dumping indicating that H2Av is lost from nurse cell nuclei. f) The mean cytoplasmic intensity of the nurse cells indicates an initial increase followed by a decline in H2Av nurse cell cytoplasmic concentration. g) The intensity in the anterior oocyte shows an initial dramatic increase followed by a slight decline (h) The intensity in the posterior oocyte is initially flat and increases only later during dumping. The accumulation is delayed compared with the anterior oocyte in Fig. 4g indicating an overall posterior flow. Images in (Fig. 5a and c) were processed for presentation using background subtraction, Gaussian filter, and median filters in Fiji.

An Arduino (Arduino, 7630049200623) was programmed to open a solenoid valve (Plum Garden, PL-220101) via a relay (TOLAKO, BJ-DT0Y) on receiving a trigger signal from the microscope for 4 min. All time-lapse imaging experiments were conducted on an inverted Zeiss LSM980 microscope controlled by Zen blue (Carl Zeiss, Zen version 3.6). Zen's Experiment Designer module was used to coordinate trigger-out signals via an external trigger box (Carl Zeiss, User Interface Box 1437–440) to the Arduino. This module also allowed us to introduce imaging pause and acquisition intervals. At the start of the experiment, we set a trigger-out signal to Arduino. Upon receiving the signal from the microscope, the Arduino opened the solenoid valve for 4 min allowing CO_2_ flow into the sample dish. After sending out the trigger-out signal, we set a 2-min imaging pause following CO_2_ inflow. This pause helps the flies succumb to anesthesia before image acquisition. We limited the duration of image acquisition to 2 min to correspond to the anesthesia time. Following the completion of imaging, the CO_2_ was withdrawn. Imaging intervals were set according to the experimental requirements (2 h for growth rate measurements (Fig. 2g, Supplementary Fig. 2d, Fig. 3c and e), 1 h for assessing effect of imaging intervals and yolk protein uptake and (Supplementary Fig. 1b and d, Fig. 4a) and 10–11 min for assessing effect of imaging intervals and H2Av-mRFP transfer experiment (Supplementary Fig. 1b and c, Fig. 5a and c).

### Microscopy

All the time-lapse imaging was conducted on Zeiss LSM980 confocal microscope with Airyscan. The system is equipped with 4 lasers (405, 488, 561, and 633 nm), 2-multi-alkali PMT detectors, a GaAsP detector, and a motorized Axio Observer-7 stage with a *z*-piezo insert. Images were acquired in Zeiss Multiplex mode (Airyscan MPLX CO-8Y) with either 10× (0.5 NA) air objective or a 20× (0.8 NA) air objective. We were able to reach a *z*-depth of ∼100 µm with a *z*-step size ranging from 3 to 5 µm. We utilized the fastest mode of acquisition with pixel time range 0.34–0.56 µs for 1–2, fluorophore with pixel sizes ranging from 0.274 × 0.274 × 5 µm to 0.149 × 0.149 × 4 µm, respectively. Further details of pixel size, pixel time, fluorophores used, laser setting, and imaging intervals are described for each experiment in Supplementary Table 2.

Egg chambers used for 3D reconstructions (Fig. 2c and d) were acquired using an upright Olympus FVMPERS multiphoton microscope equipped with the InSight DeepSee Laser System, multi-alkali photomultipliers, 2 GaAsP detectors, Olympus 25× water immersion objective (XLPLN25×WMP2, NA 1.05 531) running FluoView software. We used 920 and 1,040 nm lasers for GFP and mRFP, respectively. We imaged to a *z*-depth of 110 µm with 3 µm step size. Note this microscope was not equipped for pulsed anesthesia and used to acquire only single time frame images (Fig. 2c–e′).

Brightfield images and recordings of whole flies in the mounts (Fig. 1c and d, Supplementary Videos 1–3) were captured using a digital USB-pluggable 60×-250× Digital microscope (Plugable, USB2-MICRO-250X).

### Fixed egg chamber sample preparation

Ovaries from 5 to 7 females were dissected in 0.1% PBS-Tween and fixed in a 1:1 mixture of 4% formaldehyde (Thermo Scientific, 28908) and heptane (Sigma Aldrich, 34873) for 20 min at room temperature. After fixation, samples were washed 3 times for 10 min each in 0.1% PBS-Tween. These processed samples were then mounted in a 1:1 mixture of Aqua-Poly/Mount (Polysciences, 18606) and RapiClear (SUNJin Lab, RC147001) mounting media. The experiment was performed twice for a total *n* = 15 females. These fixed samples were used to construct a standard growth curve.

### Image quantification

*Egg chamber midsection area measurements*. Nuclear H2Av-mRFP signal from follicle cells was used to trace the midsection area of the egg chambers using the polygon tool in Fiji (Version 2.14.0/1.54f) (Jia *et al*. 2016).

*Staging fixed egg chambers.* Where possible we used size-independent criteria to stage egg chambers. Due to lack of suitable markers, egg chambers younger than stage 4 were distinguished based on their size. The chromosome condensation status, which changes from blob-like to dispersed, was used to distinguish stages 4, 5, and 6. Oocyte nuclear position, which moves anteriorly, distinguished stage 6 from 7. Increased proportion of the oocyte area distinguished stages 8–11 along with border cell migration and nurse cell dumping. By the completion of stage 8, the oocyte accounts for 30% of the egg chamber area. The initiation of the border cell migration is an indication of stage 9 and the completion of migration identified stage 10. The onset of dumping at stage 11 brings about a dramatic increase in the oocyte size, whereas stages 12 and 13 were distinguished based on the appearance of dorsal appendages. Stage 14 is marked by the complete clearance of the nurse cell nuclei (King 1970). We staged 234 fixed egg chambers to calculate the frequency distribution of different stages of egg chambers. Further, we also counted the number of egg chambers in each ovariole from the fixed samples (mean number of egg chambers/ovariole (EO) = 5.2 ± 0.09 SEM; *n* = 45).

*Estimating oogenesis timeline to generate growth curve.* We counted the number of ovarioles in a female by dissecting the ovaries from well-fed 4–5 days old, mated females in 0.3% PBS-tween. We scored 16 females from 2 independent replicates (mean number of ovarioles per female(O) = 35.63 ± 0.46 SEM, *n* = 16). To obtain the average number of eggs laid per female per day, we maintained 4–5-day-old females and males in individual pairs in fresh food vials supplemented with fresh yeast paste and recorded the number of eggs laid by individual females in 24 h. We recorded the fecundity of 8 females from 2 replicates (mean number of eggs laid per female/day (E) = 51.5 ± 2.65 SEM; *n* = 8). This gave us an egg production rate per ovariole (EPR = E/O) (David and Merle 1968; King 1970; Spradling 1993) of 1.45 eggs per day by an individual ovariole (EPR = E/O). From this, we estimated the total time required for progression from stage 2 to stage 14 (G = EO/EPR) as 86h. Finally, we calculated the growth duration of an egg chamber at a specific stage (Gs) based on the frequency with which it occurred in our samples (e.g. since 7.7% of our egg chambers were in stage 8, Gs_8_ = 6.65 h). The frequency of distribution for all stages and their corresponding development time is given in Supplementary Table 1.

With the estimated duration for the individual stage and the midsection area, we fit a standard exponential growth curve: A = 273 e^0.06t^, where *t* = time in hours, A = area in µm^2^. From this equation, we calculate the expected time t_0_ (initial age of the egg chamber at the being of the imaging session) for the known area measurements for each time-lapse image in Fig. 2g, Supplementary Fig. 2d and d″ and Fig. 3c–f.

We measured the fraction of the egg chamber area occupied by the oocyte from a manually segmented single midsection frame for each oocyte (Supplementary Fig. 3b). We generated a standard exponential growth curve based on oocyte percent for the stages 8–11 using measured oocyte percent and the estimated duration from fixed samples (*n* = 44). Here is the exponential equation for the oocyte percent: O = 0.318 e^0.07t^, where *t* = time in hours, O = oocyte percent to fine-tune the staging of the egg chambers for yolk uptake and histone transfer experiments (Figs. 4 and 5). From this equation, we calculated the expected time t_0_ (initial age of the egg chamber at the start of the imaging session) from the know oocyte percent of the live imaged samples of Yp1-sfGFP and H2Av-mRFP quantification experiments. We also calculated the correlation for oocyte percent and egg chamber area (Supplementary Fig. 4a).

*Yolk proteins quantification.* To quantify the sfGFP-tagged yolk protein, we drew a region of interest (ROI) around the oocyte using the H2Av-mCherry signal. Due to our inability to image the full depth of the older egg chambers, we considered one-half of the egg chambers for quantification (top through midsection). We measured the total intensity of GFP for all the *z-*slices across one-half of the egg chamber. Similarly, we measured the total intensities of H2Av-mCherry from the follicle cells to normalize the GFP intensities and obtain normalized total Yp1 intensities over the developmental duration. We also measured the amount of Yp1-sfGFP from fixed samples. The fixed egg chambers were aligned to the standard curved based on the oocyte percent similar to the Bellymount-PT samples.

*H2Av-mRFP quantifications.* To quantify the total nuclear H2Av-mRFP intensities and volume of the nurse cell nuclei (Fig. 5e; Supplementary Fig. 4c) we segmented 1–2 posterior nuclei per egg chamber in 3D using a custom-built Fiji macro, which apply Gaussian Blur in 3D (5,5,1) and set an automatic threshold based on Otsu method to generate masks. These masks were further processed by applying erode, dilate, open, close, and fill holes functions to refine the segmentation of the nuclear signal across the *z-*stacks. Processed masks were converted to ROIs using analyze particles function. These ROIs were used to measure the nuclear area and pixel intensities from unprocessed images.

To estimate how local concentrations change over time (Fig. 5b, d and f–h), we measured average pixel intensity in 20 × 20 pixel (428.5 µm^2^) regions in a set of 3 *z-*slices (step size 3 µm) starting 9 µm below the follicle cell border. Nurse cell cytoplasm was defined as a nuclear-free region next to the quantified nucleus within a distance of 100 pixels (17 µm) from the oocyte border. “Anterior oocyte cytoplasm” was defined as a nuclear-free region within 100 pixels (17 µm) from the border of nurse cells on the oocyte side. “Posterior oocyte cytoplasm” was defined as a region within 100 pixels (17 µm) from the posterior border of egg. For quantifying H2Av-mRFP overtime, we considered every fifth time frame. We manually placed the ROI at every time frame considered for analysis to ensure the ROI was placed in a relatively consistent region across the image duration since the sample moved in all 3 planes during the imaging intervals. The nurse cell nuclear and cytoplasmic and oocyte cytoplasmic intensities were normalized to follicle cell intensities. The same criteria were used to measure the H2Av-mRFP intensities in fixed samples.

*3D surface reconstruction.* H2Av-mRFP signal from the nurse cell nuclei was used to reconstruct the nuclear surfaces, while follicle cell nuclei were used to reconstruct surfaces for the whole egg chambers using the Arivis Vision4D program (version 4.0) (Fig. 2d). To improve 3D reconstruction quality, the *z-*axis was resampled using bicubic interpolation by a factor of 3, in Fiji, while maintaining the original *x*/*y* pixel dimensions. The analysis pipeline included denoising with a median filter (5 µm), followed by thresholding using the region growing function in Arivis with a watershed threshold and a maximum distance of 2 µm. Nurse cell nuclei were further segmented using the seeded region growing function with seed detection threshold of 78%. The H2Av-mRFP signal on the surface of the egg chambers was used to train the Arivis machine learning-based draw tool, and segmentation errors were manually corrected.

*Tracking follicle cells for measuring velocity and angular velocity.* A stage 8 egg chamber was analyzed to measured follicle cell velocity and angular velocity by manually tracking 5 representative cells. The egg chamber was oriented horizontally along the anterior–posterior axis so that the rotation was oriented along the *y*-axis, and the width of the egg chamber along the shorter axis in a mid-focal plane was used to determine the radius of the of the egg chamber (r). Angular displacement (θ) was calculated using: θ = arctan (y-y_mid_/r)*(180/π) degree/hour, where y = y coordinate at t_n_, y_mid_ = mid-point along the anterior–posterior axis, r is the radius of the egg chamber. Angular velocity (degree/hour) was calculated angular velocity = Δθ/Δt.

### Figure preparation

Images were processed in Fiji for figure preparation (Figs. 1f, g, 2c, d, f, g, 3c, e, 4a and 5a, c, Supplementary Figs. 2a, d, d″, 5) using the following functions: Subtract background with a radius of 50–100 pixels, Gaussian blur of radius 0.5–1 pixels, and a Median blur of radius 1–2 (Jackson *et al*. 2023). All quantifications involving fluorescents intensities were performed on unaltered images. We combine 4–7 *z-*slices from the middle of the oocyte via maximum intensity projection and registered the movies using the StackReg plugin in Fiji. For larger movements, we manually aligned the egg chamber images. To demonstrate the ability to track individual cells during egg chamber development, we manually tracked cells using H2Av-mRFP marker. Supplementary Fig. 5 compares the unprocessed and processed images. Exponential growth curves and graphical presentation of data were prepared out in Python environment. Pictorial presentations and figure panels were prepared in Adobe Illustrator (Version 27.9).

### Statistics

*P*-values for fecundity and different liquid food diet (Fig. 2b, Supplementary Fig. 1a) were derived from a 2-tailed student *t*-test using Microsoft Excel (Version 16.83). The number of biological replicates is indicated in the figure legends and text.

## Results

### Bellymount-PT integrates image acquisition with anesthesia to allow long-term imaging in the mount

To maintain long-term tissue health in the endogenous physiological context during imaging, we developed Bellymount-PT. Flies were secured between glass surface of a MatTek dish and a compression coverslip using Elmer's glue and 0.48 mm adhesive spacers (Fig. 1a). Flies were repeatedly anesthetized using CO_2_ to prevent voluntary and involuntary movements during image acquisition, CO_2_ pulsing allowed the flies to awaken and feed between timepoints (Poinapen *et al*. 2017; Koyama *et al*. 2020; Tang *et al*. 2021). Since CO_2_ anesthesia adversely affects the fertility of flies by disrupting cellular pH and progression through later stages of oogenesis (Shen *et al*. 2020; Sustar *et al*. 2023; Zimmerman and Berg 2024), we minimized the duration of anesthesia by integrating image acquisition with CO_2_ pulsing via an Arduino controller. The Arduino opened a solenoid valve on the CO_2_ tank in response to a signal from the image acquisition software (Fig. 1b) 2 min before the beginning of imaging and closed the valve immediately after image acquisition. Pulsed anesthesia allowed flies to awaken, feed, defecate, and lay eggs while restrained in the mount between image acquisitions (Fig. 1a, c, and d, Supplementary Video 1). We tracked egg chambers with imaging intervals from 10 min to 2 h depending on the experimental requirements.

Up to 5 flies can be mounted on a single 50 mm dish (Fig. 1b and d, Supplementary Video 2). We typically imaged 3–4 flies during each session and the unimaged flies served as controls to determine the effects of laser exposure on fly health (Fig. 2a and b). This ability to image multiple flies in a single experiment will help to control for day-to-day variability when multiple genotypes are imaged simultaneously. Since oogenesis is extremely sensitive to the nutritional status of the female (Terashima and Bownes 2004; Shimada *et al*. 2011), we developed a custom apple juice and yeast extract liquid food which was provided via a cotton wick in a bent capillary feeder tube (Fig. 1a, Supplementary Fig. 1a). To stimulate olfactory feeding cues, we included fresh yeast paste and banana baby food within the reach of each fly's legs. The food and a moist absorbent pad positioned at the top of the imaging chamber kept the flies hydrated during imaging (Fig. 1a–d).

### Females mounted for Bellymount-PT remain alive and fecund

Pulsing CO_2_ enables us to limit the exposure to anesthesia to the minimal period during image acquisition. Providing continuous access to food with an imaging interval of 2 h allowed ∼60% of flies to survive for >16 h of imaging (Fig. 2a, Supplementary Fig. 1b). To understand the causes of mortality during imaging, we tested the effects of restraint, CO_2_, and image acquisition frequency. We maintained controls on standard food in vials, in the mount without exposure to CO_2_ or laser, on the microscope exposed to repeated anesthesia but not imaged, and imaged at 10 min, 1 h, and 2 h interval (Fig. 2a, Supplementary Fig. 1b). As expected, nearly all flies maintained in vials survived and flies restrained in the mount and exposed to both laser and CO_2_ had the highest mortality in all cases. Increasing the imaging frequency from 2 to 1 h did not meaningfully reduce survival, but flies imaged every 10 min experienced a 100% mortality rate after 16 h (Supplementary Fig. 1b and Video 8). Nonetheless, egg chambers imaged at 10-min intervals frequently continue to develop for 6–8 h (Supplementary Video 7). The bulk of the mortality seems to be due to the restraint system itself since the flies in the mount that were not exposed to anesthesia, or the laser had only about a 10% increase in survival compared with those that were imaged every 2 h (Fig. 2a, Supplementary Fig. 1b). We observed that for flies that were imaged once every 1 or 2 h much of the mortality resulted from dehydration or drowning when the wick for the liquid food was not ideally placed above the fly. At 10-min imaging intervals, desiccation became increasingly apparent.

Fecundity is a crude reflection of the rate of egg chamber development. To understand the influence of experimental conditions on egg production, we counted the eggs laid by females imaged during an overnight (16 h) session, along with other controls as described above. Restrained flies frequently continued to lay eggs, which accumulated in the mount at the posterior of the fly (Fig. 1c, Supplementary Videos 1–3). On average, flies that remained in their food vials at 19–20°C laid 10 ± 2 (SEM) (*n* = 37), while flies that were imaged every 2 h laid 3 ± 1 (SEM) eggs per female overnight (*n* = 59) (Fig. 2b). More frequent imaging further reduced fecundity (Supplementary Fig. 1c and d). Although restraint did significantly reduce egg production (*P* < 0.0001), the fact that egg production continues even during imaging indicates that oogenesis is not completely stalled under the Bellymount-PT protocol.

When stressed, flies will often reabsorb young egg chambers resulting in a lower overall egg production rate. To further assess egg chamber health during the Bellymount-PT protocol, we measured the number of degenerating egg chambers immediately after mounting compared with after 6 h of imaging. We found that the percentage of degrading egg chambers increased from ∼10% at *t*_0_ to ∼30% at 6 h indicating a meaningful increase in egg chamber degradation over the imaging period (Supplementary Fig. 2a–c). Nonetheless, even after 6 h of imaging the majority of egg chambers did not show visible signs of deterioration. We note that initial fly health is predictive of egg chamber degradation rates. Files with more unhealthy egg chamber at the beginning of the imaging session had higher rates of degeneration after 6 h (Supplementary Fig. 2c).

### Imaging through the cuticle allows single-cell resolution of developing egg chambers

Bellymount-PT allows the identification of all stages of oogenesis from the germarium through stage 14 using H2Av-mRFP, although not all stages were visible in the same female (Fig. 1e–g). While larger later-stage egg chambers were easier to locate, we frequently observed the germarium and early-stage egg chambers. The precise imaging parameters required depend on the resolution and duration of imaging required for a specific experiment. We can easily resolve individual H2Av-mRFP nuclei and Moesin-GFP at the cortex within egg chambers to a z-depth of 75–80 µm using an Olympus multiphoton microscope with a 25× water objective (NA 1.05) (Fig. 2c). The nuclear signal of follicle cells defines the outer egg chamber boundary allowing for accurate 3D reconstruction of full egg chamber volumes up to stage 8 (stage 6 shown in Fig. 2d). Later-stage egg chambers could not be imaged to their full depth, but in all cases, the midsection area functions as a proxy for egg chamber size. We can resolve subcellular structures, including ring canals and oocyte chromatin (Fig. 2c–e″, Supplementary Videos 4–6). At lower magnification (Zeiss 980 with a 10× NA0.5 air objective) several egg chambers of different ages can be imaged simultaneously (Fig. 2g, Supplementary Video 9) increasing the throughput of the technique. The wider range of view of the lower magnification objective also aided in long-term tracking since egg chambers move within the female abdomen throughout development and the female herself moves a small amount within the restraints during imaging intervals. Under these conditions, we can track multiple egg chambers through different stages of development for 8–16 h (stage 10–14, Fig. 2g, Supplementary Fig. 2d and d’, Supplementary Videos 9 and 10; stage 7–8, Fig. 3c and e, Supplementary Videos 11 and 12). At 20× magnification, we were able to manually track individual follicle cells of a stage 8 egg chamber imaged every 10 min for ∼3 h (Supplementary Video 13). These cells had an average velocity of 0.56 µm/minute and angular velocity of 28.5 degrees/hour, which are comparable to previous measurements from cultured egg chambers (Chen *et al*. 2016; Ku *et al*. 2023). At the same magnification, we were also able to track follicle cell movements during dorsal appendage formation (Supplementary Video 14).

### Bellymount-PT enables tracking live growth rates of individual egg chambers

Due to the challenges of live-imaging oogenesis, existing estimates of egg chamber growth rates are based on the number of egg chambers of each stage in individual females at a given time and the average number of eggs laid per female per day. Moreover, literature estimates differ greatly in the time assigned to each stage due to differences in genetic backgrounds and fly husbandry (David and Merle 1968; King 1970; Spradling 1993). To account for our own strains and fly husbandry, we generated a new growth curve for the developmental time of w; Mat-ɑ-tub67-gal4/CyO; Mat-ɑ-tub15-gal4, H2Av-mRFP/TM3 females maintained at 23 ± 1°C (Hunter and Wieschaus 2000). Note that our standard growth curve is constructed from flies that were kept at warmer temperatures than our imaging conditions. Since the conventional staging of egg chambers is largely based on size and we wanted to measure growth rates, we staged the egg chambers based on size-independent parameters whenever possible and measured the egg chamber midsection area for each stage from the fixed samples (*n* = 15 females; *n* = 234 egg chambers) (Fig. 3a, Supplementary Fig. 3a; see methods). We recorded the fecundity (eggs laid/female/day = 52 ± 2.7 SEM), egg production rate (1.45 eggs/per ovariole/day), and the frequency of each stage in an ovariole to estimate the duration of each stage 2–14 (Supplementary Table 1). This data allowed us to estimate the expected growth rate for egg chambers during unperturbed oogenesis, which is well fit by an exponential equation (A = 273 e^0.06t^, where *t* = time in hours and *A* = area in µm^2^). Our measured standard growth curve is within the range reported in previous literature (King 1970; Spradling 1993) (Fig. 3a).

Next, we sought to measure the real-time growth rate of individual egg chambers inside the female abdomen using Bellymount-PT. We were able to track the growth of egg chambers, which were initially in stages 4–10. We measured the midsection area of each egg chamber over time. To test if the growth rates observed using Bellymount-PT matched those expected from the fixed growth curve, we aligned each trace to the growth curve based on the initial area with the subsequent times based on the imaging intervals. While some egg chambers had growth rates that aligned well with the expected growth curves (Fig. 3b–d, Supplementary Video 11), most grew more slowly than our standard (Fig. 3b, e, and f, Supplementary Video 12). During the initial 2 h of imaging, the imaged egg chambers reached an average of 95% of the expected size, however by 8 h, they had only reached 72% (Supplementary Fig. 3c). We note that although growth rates decreased, many of the size-independent aspects of oogenesis progressed at a relatively normal rate indicating that the egg chambers continued to develop (Fig. 3b, Supplementary Fig. 3b). This suggests that the midsection area may be an underestimate of egg chamber volume due to compression by neighboring chambers which may reduce the cross section in x/y and increase the volume in z. In particular, stage 10 egg chambers were often seen to progress to stage 14 in ∼10 h as expected but did not appear to grow as much as expected (Fig. 2g, Supplementary Fig. 2d and d′). To further assess egg chamber development rates, we analyzed the percentage of the egg chamber area occupied by the oocyte over time for egg chambers of different starting sizes. (Supplementary Fig. 3b). We found that the percentage oocyte area increased in the selected egg chambers in a manner similar to fixed egg chambers from control flies Fig. 3b.

### Bellymount-PT allows unprecedented visualization of the events of oogenesis

Finally, we employed Bellymount-PT to capture dynamic protein localization events during oogenesis. First, we focused on the uptake of yolk protein into the oocyte from the hemolymph. The initiation of yolk uptake is a checkpoint for further progression of the egg chamber development. Active yolk uptake starts from early stage 8 and continues through stage 10 (Terashima and Bownes 2004; Shimada *et al*. 2011; Isasti-Sanchez *et al*. 2021; Row *et al*. 2021). Inadequate nutrition and environmental stress stall oogenesis at stages 7 or early 8 by preventing yolk uptake (Terashima and Bownes 2004; Shimada *et al*. 2011; Hara and Yamamoto 2022). *Drosophila* yolk proteins (Yp1-3) are predominantly made in fat bodies and ovarian follicle cells, and their endocytosis by the oocyte is stimulated by factors including hormonal levels (Richard *et al*. 1998) and male-derived sex peptide received during mating (Soller *et al*. 2006). Thus, yolk accumulation requires the coordination of multiple organ systems and external factors. This explains the difficulty in maintaining egg chamber growth during these active vitellogenic stages in ex vivo culture systems (Peters and Berg 2016). To test if Bellymount-PT supports yolk uptake, we tracked Yp1:sfGFP (Hara and Yamamoto 2022) accumulation in stage 8–10 egg chambers (*n* = 6). Since in later stages growth is dependent on yolk uptake, we aligned the growth curves to the expected initial age based on the initial oocyte percentage instead of the midsection area (Fig. 4c). We used an endogenously tagged H2Av-mCherry (Shindo *et al*. 2024) to manually segment the oocyte and measured the total amount of Yp1-sfGFP protein in ½ of the egg chambers over time. We found that yolk continually accumulates at a relatively constant rate for the duration of the measured stages (Fig. 4a and b, Supplementary Video 15). The pattern of Yp1-sfGFP accumulation observed by Bellymount-PT was similar to that seen in the fixed egg chambers matched by oocyte percentage (Fig. 4b). These direct measurements of yolk uptake from the hemolymph are only possible by preserving the physiological context inside the intact female abdomen using Bellymount-PT.

We were interested in measuring maternal protein transfer from the nurse cells to the oocyte during dumping. Histones are among the essential nuclear components that are loaded into the oocyte during nurse cell dumping to support the early embryonic cell cycles. The oocyte is unusual in that it generates large stores of both replication-dependent and replication-independent histones, which are stable beyond S-phase (Li *et al*. 2012). Maternal histone loading is of great importance, as histones concentration regulates the progression of early embryonic development (Amodeo *et al*. 2015; Chari *et al*. 2019; Shindo and Amodeo 2019; Shindo and Amodeo 2021). Like most proteins, replication-dependent histones are synthesized in the nurse cells and transferred to the oocyte during the later stages of oogenesis (Ambrosio and Schedl 1985; Ruddell and Jacobs-Lorena 1985; Walker and Bownes 1998). However, a recent study showed that H2Av is transferred to the oocyte as early as stage 9 (Stephenson *et al*. 2021), suggesting that different histones can behave differently during oogenesis.

To measure the flow of histones into the oocyte we measured the concentration of H2Av-mRFP in various regions of the egg chamber during nurse cell dumping (Fig. 5). We also segmented the nurse cell nuclei to measure how the nuclear volume and total H2Av-mRFP changed over time (Supplementary Fig. 4c, Fig. 5e). We found that the nurse cell nuclei lost H2Av-mRFP over time (Supplementary Fig. 4c  Fig. 5e). However, nuclear volume decreased more rapidly than the H2Av-mRFP loss resulting in a transient increased nuclear concentration (Supplementary Fig. 4b and c). At the same time, the cytoplasmic intensities of H2Av-mRFP increased in the nurse cells and the anterior and posterior regions of the oocyte with the anterior preceding the posterior (Fig. 5a and b, Supplementary Video 16). After completion of dumping, we observed a drop in H2Av-mRFP concentrations both in the anterior and posterior regions of the oocyte (Fig. 5c and d, Supplementary Videos 17–19), likely due to the dynamic streaming of the oocyte cytoplasm and an increase in the overall volume of the oocyte. We also measured the dynamics of H2Av-mRFP from fixed samples and found a very high level of egg-chamber to egg-chamber variability, which highlights the importance of capturing these dynamics in vivo. Therefore, Bellymount-PT enabled us to capture the dynamic flux of the maternal proteins in real time during the later stages of oogenesis.

## Discussion

Bellymount-PT provides a powerful means to capture dynamic processes in living animals with minimal perturbation. We have focused on *Drosophila* oogenesis, which is intricately regulated by organismal signals, including nutritional availability, mating status, and environmental conditions. While egg chambers can survive in ex vivo culture for a limited time, these systems are unable to faithfully replicate the physiological growth cues, signaling events, and stress response systems found in vivo. The noninvasive nature of Bellymount-PT allows access to internal, subcellular details while preserving the native physiological milieu. We can capture all stages of oogenesis, including the germarium and track multiple egg chambers for over 16 h, observing progressive developmental stages. The ability to image vitellogenesis, a critical checkpoint in oogenesis that is difficult to study in cultured systems, demonstrates the improved egg chamber health compared with other state of the art techniques.

One notable constraint of Bellymount-PT is the inability to capture the entire length of oogenesis with normal growth rates. We observed a ∼40% mortality rate in flies imaged once every 2 h and decreased fecundity compared with unrestrained flies. More frequent imaging, as would be required to capture rapid events such as boarder cell migration (Prasad *et al*. 2015), is possible with a minimum of 4 min as required to anesthetize the fly. However, imaging frequencies of 10-min drastically reduce long-term survival in the mount. Two major causes of mortality were drowning in the liquid food and desiccation within the mounts. Further optimization of the feeding protocol may improve fly health and thereby increase the overall rate of egg chamber development. Another hurdle for fly health during imaging is presented by the common practice of housing microscopes in cool, dark rooms. Increasing the temperature and scheduling imaging sessions to align with the fly circadian rhythm would also likely further increase fecundity. Nonetheless, Bellymount-PT represents a significant improvement in the length of egg chamber growth and survival over existing techniques and allows for the capture of rapid events such as nurse cell dumping. Bellymount-PT can be easily implemented in any laboratory equipped with a confocal microscope without requiring specialized equipment. The capability to image multiple females simultaneously increases the throughput and allows multiple genotypes to be imaged under identical conditions.

The utility of Bellymount-PT extends beyond oogenesis. Indeed, the original Bellymount protocol was developed to capture intestinal stem cell dynamics. Within the gut, Bellymount has sufficient resolution to differentiate individual bacteria (Koyama *et al*. 2020). Bellymount-PT is compatible with the long-term imaging of other abdominal tissues such as the crop, tracheal tubes, fat bodies, and blood cells. Therefore, Bellymount-PT presents an attractive alternative to current ex vivo culture modalities, especially for processes such as yolk uptake that require coordination across multiple tissues.

## Data Availability

All relevant data can be found within the article and its supplementary information. Supplementary information including tables, figures, and videos can be accessed through gsajournals.figshare.com at https://doi.org/10.25387/g3.27626058. The code for CO_2_ pulsing and imaging analysis is available at https://github.com/Shrchandra/SB_AA_Bellymount-PT. Supplemental material available at G3 online.
